# The associations between ADHD, pain, inflammation, and quality of life in children and adolescents—a clinical study protocol

**DOI:** 10.1371/journal.pone.0273653

**Published:** 2022-09-09

**Authors:** Nóra Kerekes, Sara Lundqvist, Elke Schubert Hjalmarsson, Åsa Torinsson Naluai, Anne-Katrin Kantzer, Rajna Knez

**Affiliations:** 1 Department of Health Sciences, University West, Trollhättan, Sweden; 2 Child and Adolescent Psychiatry, Queen Silvia Children’s Hospital, Gothenburg, Sweden; 3 Institute of Neuroscience and Physiology, Sahlgrenska Academy at the University of Gothenburg, Gothenburg, Sweden; 4 Department of Physiotherapy, Queen Silvia Children’s Hospital, Sahlgrenska University Hospital, Gothenburg, Sweden; 5 Institute of Biomedicine, Sahlgrenska Academy at the University of Gothenburg, Gothenburg, Sweden; 6 Child and Adolescent Psychiatry, NU Hospital Group, Trollhättan, Sweden; 7 Department of Pediatrics, Skaraborg Hospital, Skövde, Sweden; Universitat de les Illes Balears, SPAIN

## Abstract

New research shows that the prevalence of neurodevelopmental disorders, such as attention-deficit/hyperactivity disorder (ADHD), is increased in children and adolescents as well as in adults with chronic pain, compared to those without chronic pain. Children and adolescents with ADHD also have an increased incidence of various physical conditions associated with pain, and they more frequently suffer from inflammatory diseases. Moreover, parents of children with ADHD can often suffer from pain conditions. These epidemiological and clinical observations form the scientific basis of our study, which aims to map the relationships between ADHD, altered pain experiences/central sensitization, and inflammation in children and adolescents. We will investigate the presence of central sensitization in children and adolescents with newly diagnosed ADHD and compare it with those who have not been diagnosed with ADHD. Participants (and their biological parents) will complete surveys about their somatic health, pain experience, and quality of life. Biological samples (saliva and stool) will be collected, aiming to utilize proteome and metabolome data to discover disease mechanisms and to predict, prevent and treat them. The results from our investigation should enable an expanded understanding of the pathophysiology behind both ADHD and pain/central sensitization. Presently, there are no established protocols for addressing psychiatric symptoms when examining patients with pain conditions in a somatic care setting, nor is there any knowledge of offering patients with ADHD or other neurodevelopmental disorders adapted treatments for pain conditions. Our results, therefore, can contribute to the development of new treatment strategies for pathological pain conditions in children and adolescents with ADHD. They may also increase awareness about and provide opportunities for the treatment of attention and impulse control problems in children and adolescents with pain syndromes.

## Introduction

Attention-deficit/hyperactivity disorder (ADHD) is recognized by its core symptoms—inattention, hyperactivity, and impulsivity. There are three recognized subtypes of ADHD: predominantly inattentive, predominantly hyperactive/impulsive, and the combination of both. The lifetime prevalence of ADHD is approximately 5% [1].

ADHD is a neurodevelopmental disorder; it has a neurobiological background that affects children’s behavior and functioning during early development [2]. The underlying neurobiological background of ADHD has not yet been fully elucidated, but it has been proven to include both morphological and functional differences in the brains of patients with ADHD as compared to those without [3]. Many brain regions (e.g., cerebellum, locus coeruleus, sensory motor cortex) and neurotransmitters that are relevant to ADHD are also immersed in the pain neuronal circuit [4]. One theory is that dopamine dysregulation, which plays a role in the neurobiology of ADHD [5] and the processing of pain [6], is linked to altered pain sensitivity in patients with ADHD. Another theory suggests that dysregulation of the opioid system is implicated in both the perception of pain and the regulation of impulsivity [7].

New research shows that the prevalence of neurodevelopmental disorders, such as ADHD, is increased in children and adolescents with chronic pain [8]. Children and adolescents with ADHD have a higher prevalence of various physical pain conditions (e.g., headache, migraine, stomach pain) as well as an increased frequency of inflammatory diseases, such as asthma [9] and atopic dermatitis [10]. Clinical observation shows that parents of children with ADHD often suffer from chronic pain. This is pertinent because ADHD is a largely hereditary condition. Clinical studies show that adults with pathological pain conditions, such as fibromyalgia, have a very high prevalence of ADHD [11]. These findings confirm the relevance of the hypothesis about possible common neurobiological mechanisms behind the coexistence of ADHD and pain. In fact, more studies have investigated pain processing in psychiatric conditions, and the quality of life for individuals with other psychiatric conditions, one of which is autism spectrum disorder (ASD), another neurodevelopmental condition often co-occurring with ADHD. However, findings regarding the alteration of pain perception in patients with ASD are conflicting, with some studies reporting hyperalgesia and others reporting hypoalgesia [12].

Today’s pain research highlights the concept of “central sensitization” (CS), or the brain processing both internal and external stimuli with increased sensitivity [13]. Our hypothesis is that children/adolescents with ADHD have altered perceptions of pain that can be detected clinically as signs of CS. Some previous studies have been performed on this subject, but their results have been difficult to interpret, and the methods for measuring pain sensitivity have varied. Smaller studies are available in which increased pain sensitivity has been detected in adults with ADHD [14].

Multiple lines of evidence (preclinical, clinical genetics, and bioinformatics) show that the activation of immune system molecules and pathways can contribute to and play a significant role in the pathogenesis of psychiatric disorders [15–21]. Some studies have investigated the role of neuroinflammation in ADHD [22–24]. One systematic review suggested that concentrations of interleukins (ILs)—IL-13, IL-16 (anti-inflammatory cytokines), IL-6, IL-10, and IL-8—were increased in ADHD patients, increasing the risk of attention problems [23].

Epidemiological studies, including meta-analyses, have revealed that patients with ADHD are more likely to suffer from asthma, allergic rhinitis, atopic dermatitis, and allergic conjunctivitis—all well-known inflammatory conditions—than control subjects. Associations between ADHD and systemic autoimmune diseases (allergies and atopic diseases) have also been shown [25, 26].

Assuming inflammation plays a key role in the pathogenesis of psychiatric disorders, anti-inflammatory treatments may represent a promising therapeutic intervention.

In the present project, we aim to 1) gather clinical data regarding the association between ADHD (with or without other co-occurring conditions) and pain and 2) suggest neuroinflammation as a possible link between altered pain perception and ADHD (and co-occurring conditions) and, therefore, that targeting neuroinflammation may be a promising therapy [4]. The Pain in Psychiatric Conditions (PiPCo) project [27] comprises both preclinical and clinical studies.

The clinical portion of the project aims to investigate subjective pain experiences and pain perceptions in children and adolescents with newly diagnosed ADHD while monitoring their inflammatory biological markers. More specifically, we propose to:

1. Investigate whether subjective pain experiences (by self-reported and parent-reported inventories) and pain perceptions (CS detected by sensitivity to pressure) differ between groups of children with ADHD only, with ADHD+autism spectrum disorder (ASD), with ADHD+other co-occurring psychiatric conditions, and without ADHD.
2. Determine whether levels of inflammatory markers (C-reactive protein [CRP], lactoferrin, cortisol, IL-6, IL-10, tumor necrosis factor [TNF]-alpha, IL-1beta, and calprotectin) differ between the aforementioned groups.
3. Investigate functional impairment and quality of life in children and adolescents with and without ADHD and with and without co-occurring psychiatric conditions in correlation with pain experience and perception.

## Study protocol/methods

The study protocol was developed in accordance with the SPIRIT (Standard Protocol Items: Recommendations for Interventional Trials) guidelines and checklist (https://www.spirit-statement.org/). Participants will be recruited to the clinical group from child and adolescent psychiatric (CAP) clinics in the Västra Götaland region of Sweden and to the comparison group from schools in the Västra Götaland region. Inclusion criteria for the clinical group include 1) fluently Swedish-speaking child/adolescent (age 7–18) and at least one parent and 2) a child/adolescent ADHD diagnosis within the previous 12 months. We chose to require a biological parent (not a guardian) in the study to ensure the genetic relationship between the child/adolescent and the adult—this will allow us to analyze the adults’ responses about their own pain experiences (central sensitization inventory) in correlation with their children’s responses, assuming a shared genetic component behind these responses. Exclusion criteria for the clinical group include intellectual disabilities, psychotic disorders, bipolar disorder, anorexia nervosa, bulimia nervosa, substance use disorder, and suicidal tendencies. Inclusion criteria for the comparison group include fluently Swedish-speaking child/adolescent (age 7–18) and at least one parent. Exclusion criteria for the comparison group include all aforementioned exclusion criteria for the clinical group plus an ADHD diagnosis.

### Study population

**Fig 1:**
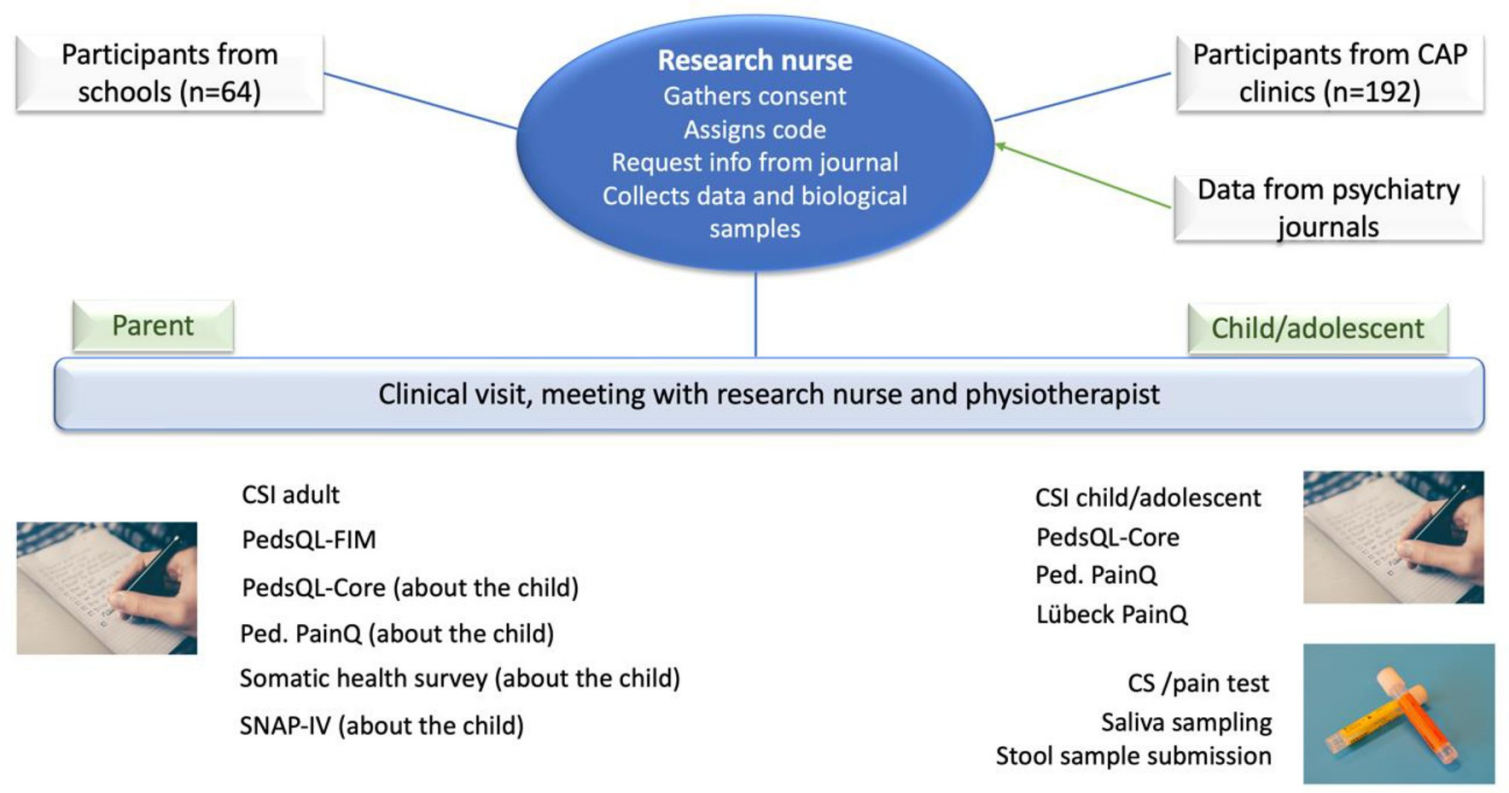
Flow chart for process description. CAP = child and adolescent psychiatry; CS = central sensitization; CSI = Central Sensitization Inventory; PedsQL-Core = Pediatric Quality of Life Inventory; PedsQL-FIM = Pediatric Quality of Life Inventory–Family Impact Module; Ped. PainQ = Pediatric Pain Questionnaire; n = by power analysis, estimated number of participants; SNAP-IV = Swanson, Nolan & Pelham Form-revised.

Parents of children and adolescents (15 years and older) who agree to participate in the study will send their written consent to a research nurse, who will pseudo-anonymize the patients in the clinical and control groups by assigning a code to each child/adolescent. For the clinical group, the research nurse will collect information about each patient’s medical record (concerning psychiatric diagnoses and drug treatment) from the CAP’s medical record system. For both groups, the research nurse will arrange a meeting at a CAP clinic in the Västra Götaland region for the participant, a parent, and a physiotherapist connected to the project (**Fig 1**). The research nurse will examine the participants; measure pulse, blood pressure, weight, and height; collect biological (saliva and stool) samples; provide them with the questionnaires; and assist them if help is required. Finally, the physiotherapist will identify signs of altered pain processing (central sensitization). During this examination of the participant, the parent will complete assigned questionnaires. The whole meeting will take approximately 60 minutes.

### Measures

All measurement procedures described below will be performed in the same manner for the clinical and comparison groups.

### Questionnaires

#### 1. Psychiatric measure

The SNAP-IV–rating scale is a revision of the Swanson, Nolan & Pelham Form (SNAP), [28]. The form includes 28 questions based on DSM-IV [29]. It assesses ADHD with two subsets of symptoms with nine questions each: attention deficit disorder (questions 1–9), hyperactivity / impulsivity (questions 11–19). Also included are eight questions for defiance syndrome (questions 21–28), as this is common in children with ADHD. Two questions have been added to summarize the two components of ADHD, attention deficit disorder (question 10) and hyperactivity / impulsivity (question 20).

#### 2. Quality of life

*The Pediatric Quality of Life Inventory–Family Impact Module* for parents is a self-assessment of parents’ and families’ feelings. It is a 36-question inventory divided into eight scales: physical, emotional, social, and cognitive function and communication, worry, daily activities, and family relationships [30]. Higher scores indicate better functioning in the family or fewer negative consequences of the child’s chronic condition [30]. The Swedish version of this instrument has been previously validated [31].

*The Pediatric Quality of Life Inventory* [32] for parents, adolescents, and children considers the child/adolescent’s health-related quality of life. The scale is available for teenagers (13–18 years) and children (5–7 and 8–12 years). The children/youth estimate their own function and well-being, and in a parallel survey, the parents estimate the same things regarding their children [33]. It is a 23-item questionnaire with four scales: physical, emotional, social, and school functioning. Higher scores on the estimate indicate better health-related quality of life. The psychometric properties of the Swedish version of the PedQL have been previously tested [34].

#### 3. Central Sensitization Inventory

*The Central Sensitization Inventory* (CSI) [35] is used for self-rating of central sensitization for adults. The scale consists of two parts: the first part describes symptoms related to CS, and the second, any diagnoses possibly linked to the same phenomenon. The scale is validated in Swedish adults [35].

*The Central Sensitization Inventory for Children and Adolescents* is an adapted version of the CSI for self-assessment of central sensitization in teens and children [36]. The validation of the Swedish version of this scale is ongoing and not-yet-published works.

#### 4. Self-reported pain perception

*A somatic health form* is used to identify any physical illnesses in a child/adolescent as reported by the parents. This questionnaire has shown high test-retest reliability [37] and has been used in its Swedish version [38].

*The Lübeck Pain Screening Questionnaire* is used for children/adolescents to estimate the incidence, type, degree, and frequency of any pain [39] and has been tested in its Swedish version [8].

*The Pediatric Pain Questionnaire* [40] assesses the complexities of chronic, recurrent pain in children. Psychometric validation has shown high correlation among visual analog scale pain intensity ratings obtained from parents and children/adolescents [41]. This instrument has been translated into several languages, including Swedish, and can be accessed at www.pedsql.org.

#### 5. Pain/central sensitization test

Pain sensitivity will be assessed by measuring each child/adolescent’s pressure pain thresholds at the trapezius muscle, deltoid muscle, lower back, and tibialis anterior muscle on her/his dominant side, distributing the measuring points on the upper and lower quadrants and axially. A handheld pressure algometer (Wagner Instruments, FPX50) with a 1 cm^2^ rubber tip and an application rate of 1 kg/s will be used. The child/adolescent will report when the pressure becomes uncomfortable.

Conditional pain modulation will be used to measure endogenous pain inhibition. The subject’s perception of the pain intensity of a test stimulus will be measured before and after the addition of a conditioning stimulus. In this study, we will use cold as a conditioning stimulus and pressure as a test stimulus. The conditioning stimulus will be applied as follows: the participant will be asked to place her/his non-dominant hand in a cold pressor unit containing water maintained at a temperature of 10 ± 1°C [42, 43]. The temperature of 10 degrees is a modification that is based on recommendations after testing of the conditional pain modulation in a Swedish population [43]. The participant will be asked to leave their hand in the water for 60 seconds. This measurement is part of a current research project at Queen Silvia Children’s Hospital, Gothenburg [43]. The licensed physiotherapist responsible for this research will be the one responsible for performing the test on each participant.

The measurement of central sensitization is not expected to pose any particular risks to the participants or their parents. However, it is important to consider that the pressure pain measurement may cause mild, momentary discomfort; some participants may experience this as painful.

Based on previous evidence regarding pain modulation theories, we expect that an individual without signs of CS will respond to the conditioning stimulus (cold) with reduced sensitivity to the test stimulus (pressure). In contrast, an individual with signs of CS will have an unchanged or increased sensitivity to the test stimulus.

#### 6. Biological markers

*A saliva sample* will be collected from each patient according to the protocol at the clinic.

Despite some differences between the protein and autoantibody levels in saliva versus blood serum, saliva is an easily accessible biological fluid that partly reflects the circulating bodily fluids. Therefore, saliva has both scientific and clinical potential [44, 45].

Collecting patients’ saliva samples as part of our study protocol allows for molecular profiles (reflective of inflammatory proteins as well as other inflammatory biological markers) to be established and used as variables. These variables will serve as valuable additions to the clinical data and make it possible to stratify patients into different groups according to their genetic or biological risk scores. Inflammatory protein and metabolite panels can be assessed, as can known genetic variants involved in autoimmune disorders, chronic inflammation, and pain. We will analyze RNA, and DNA from the saliva microbiome and the host cells within the saliva fluid by microarray and next-generation sequencing (NGS). To analyze the metabolites involved in inflammation, state-of-the-art nuclear magnetic resonance (NMR) spectroscopy will be used. Inflammatory protein biomarkers (interferons, interleukins, and proinflammatory cytokines) will be investigated via antibody-based multiplex protein panels. Selected inflammatory markers (e.g., CRP, lactoferrin, cortisol, IL-6, IL-10, IL-1ß, TNF-α) will also be analyzed individually via enzyme-linked immunosorbent assays in the CAP clinic’s laboratory. Known autoantibodies against proteins in the digestive tract and nervous system that are involved in pain perception will be investigated to rule out pain that may result from autoimmune reactions (e.g., TG2 [celiac disease], CASPR2 [pediatric autoimmune encephalitis], and autoantibodies targeting voltage-gated potassium channel complexes [e.g., LGI1 and CASPR2]) [46]. Excluding patients with these autoantibodies is likely to increase the statistical power in the remaining samples and yield a more comprehensive picture of the different patient groups.

*A stool sample* collected at home 1–2 days before the visit will be submitted at the visit. When the intestine is inflamed, calprotectin is released by neutrophils. Calprotectin is not specific, but its increased level in fecal excretions is seen in conjunction with different diseases. Therefore, we also want to measure calprotectin as a marker for inflammation, and we will examine the correlations between calprotectin and other biomarkers and symptoms using information about the child/adolescent’s intestinal flora profile as measured by 16S rRNA sequencing.

### Data analysis

Data will be collected through self-reports from children and parents, from the pressure pain measurements (central sensitization) from children, from the saliva and stool samples (for inflammatory markers), and from the medical record system regarding diagnoses and medications for the clinical group. All data will be coded.

The effect size for the measurement method of central sensitization is not yet known. Calculations are ongoing in a study at Queen Silvia Children’s Hospital, but the results are not yet clear. Therefore, we have estimated the effect size as small in the following power calculation and made a theoretical calculation of the population size. When we used power analysis for “MANOVA special effects and interactions” with an effect size of 0.07 and a power of 0.80, it was suggested that the number of participants should be 252 for eight groups.

These eight groups are: 1) comparison group boys, 2) comparison group girls, 3) clinical group boys with only ADHD, 4) clinical group girls with only ADHD, 5) clinical group boys with ADHD+ASD, 6) clinical group girls with ADHD+ASD, 7) clinical group boys with ADHD+other co-occurring psychiatric conditions, and 8) clinical group girls with ADHD+other co-occurring psychiatric conditions. Other co-occurring psychiatric conditions will include anxiety disorder, depression, stress disorder (including posttraumatic stress disorder), and behavioral disorders (e.g., oppositional disorder and conduct disorder).

In the power calculation, four predictors (ADHD, ADHD+ASD, ADHD+other co-occurring psychiatric conditions, and gender) and four response variables (self-reported pain experience, test measures of CS, inflammation, and functional level) were used. This suggests that the study requires at least 32 children per group—at least 64 children (32 boys and 32 girls) for the comparison group, 64 children with only ADHD, 64 with ADHD+ASD, and 64 with ADHD+other co-occurring psychiatric conditions.

In summary, we will include 192 children in the clinical group and 64 children in the comparison group as targets for data collection.

The differences in variance between the clinical groups and the comparison group—with respect to CS results, the prevalence and intensity of pain, and quality of life (as reported by children and parents)—will be analyzed using multivariate analysis of variance. The level of parental self-rated CS will be correlated with that reported by and tested in their own child using Spearman’s correlation. The child’s measured level of CS will be correlated by multiple regression with the following independent variables/predictors: age, gender, presence (and type) or absence of psychiatric diagnoses, self-rated CS, parent-rated CS in the child, parent self-rated CS, self-rated pain sensitivity, parent-rated pain sensitivity in the child, and levels of inflammatory markers in saliva and stool samples.

**Fig 2:**
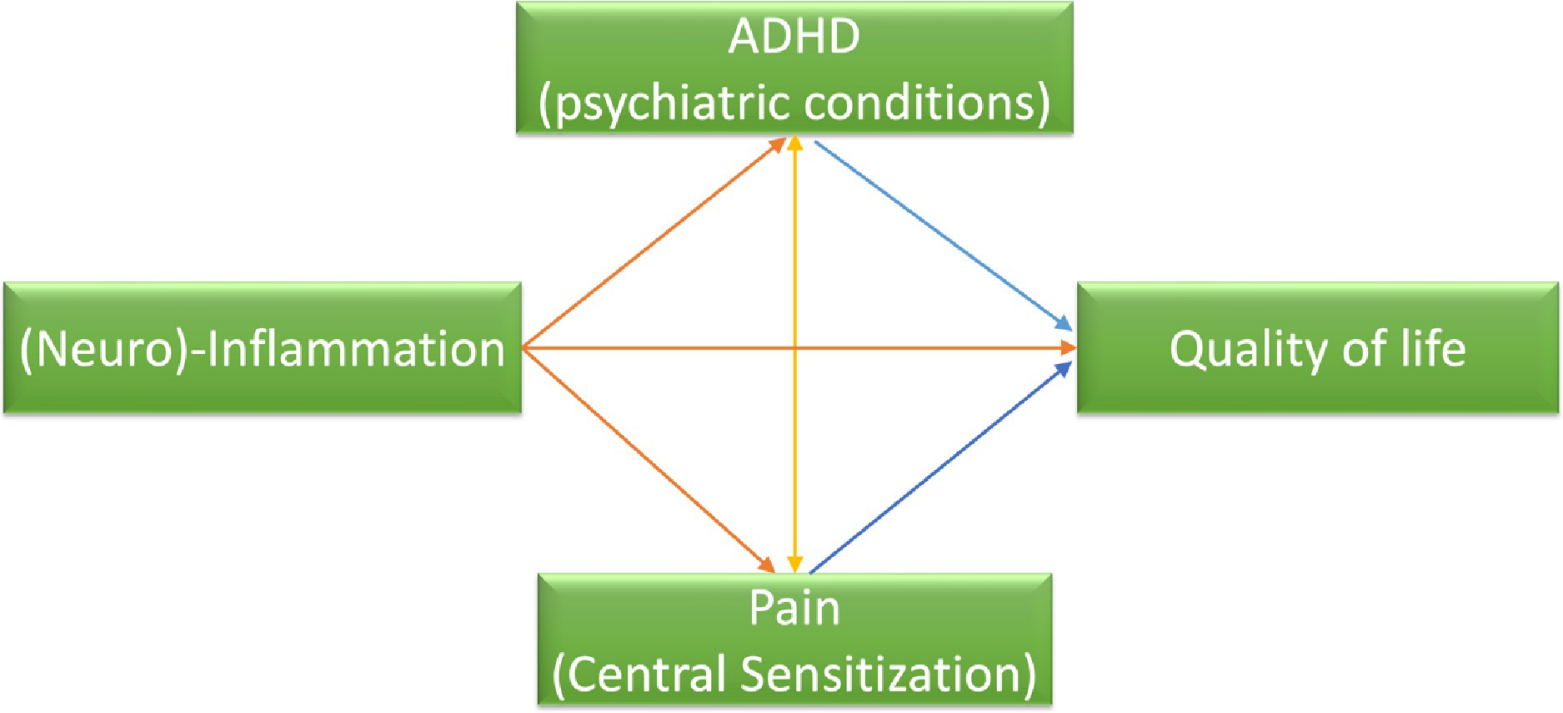
Conceptual diagram of our hypothesized model in regression-based pathway analysis.

The association between the child-reported quality of life and the parent-reported quality of life of the child and the family will be analyzed via regression models. Using conditional process analyses (regression-based path analyses by PROCESS of SPSS) (**Fig 2**), we will test a moderated mediation hypothesis between inflammation and quality of life; pain (CS) and ADHD (psychiatric conditions) will be tested as mediators and each other’s moderators (**Fig 2**).

The internal reliability of each instrument will be assessed using Cronbach’s alpha.

### Ethical considerations

The National Ethical Review Board approved the study on November 2, 2020 (protocol no. 2020–03993). The research nurse will verbally describe the study’s purpose, aim, procedure, and methods to each participant. A written form will also provide more detailed information about the background and purpose of the research, risks/benefits of participating, research structure, compensation for participation, and information about the management of project data. It will be clear that the study is voluntary and anonymous and that the data collected will only be used for the study. The participants will have the opportunity to ask questions about the project and about how their information will be used.

The instruments used in this study will be established psychiatric or physiological measures, each previously used in child and adolescent populations. They are not considered to pose any risk, either during the time of the study or in the long term.

During data collection, the clinics will follow their standardized patient safety and data security regulations. This category of data is defined as sensitive personal data according to the EU Data Protection Regulation (GDPR). Clinics participating in the study are responsible for obtaining informed written consent from the participants and then pseudonymizing (coding) sensitive personal data so that the patients’ identities are protected in an appropriate manner. Both paper-and-pen and electronic information (from clinical records) will be coded. Biological material will also be marked with the appropriate participant’s code; no personal information will be included. All samples will be destroyed upon the completion of the project. No follow-up of the participants is planned or necessary. Code keys will be available only at the clinics during data collection and will be destroyed after the coded data is transferred to University West, where data analyses will be performed. The research material will only be processed and shared with other researchers in its deidentified form.

## Results

Our research group has performed a pilot study [47] to map the prevalence of pain in children and adolescents from the CAP clinics in the Västra Götaland region. CAP patients experience pain (e.g., stomach pain, headache) more often than children without psychiatric problems. The largest difference was seen in the prevalence of stomach pain in girls with psychiatric conditions compared to girls without them (85% vs. 62%, *P* = 0.032). A similar tendency was found in boys (59% vs. 45%, *P* = 0.059). These study results are now under peer review prior to publication [47]. The subgroup of patients with neurodevelopmental disorders was overrepresented among those reporting frequent pain as compared to children with other psychiatric conditions (79% vs. 40%, *P* = 0.043).

A recent study concluded that saliva can be used to monitor ADHD status with regard to biomarkers that indicate the hypothalamus–pituitary–adrenal axis and sympathetic activity [48]. Furthermore, as previously mentioned, ADHD is associated with a higher occurrence of autoimmune and inflammatory conditions [23]. However, the evaluation of inflammatory biomarkers has provided inconsistent results thus far, possibly due to the small sample sizes and targeted biomarker approaches. Suggestive evidence still points to the possibility that increased inflammation during early development may be a risk factor for ADHD. The identification of inflammatory biomarkers, therefore, warrants further investigation, and possible biomarkers related to inflammation (as well as to pain and ADHD) can be valuable tools for future diagnosis and treatment.

Our French preclinical partner in the PiPCo study has demonstrated that their animal model of ADHD, mice with 6-hydroxydopamine (6-OH) lesions, has good constructive validity to reproduce both primary symptoms of ADHD and secondary symptoms (anxiety and antisocial and aggressive behavior) [49]. The animal model showed that pain sensitivity is increased in “ADHD mice.” This increased pain sensitivity was observed both under normal circumstances and after administration of a pain stimulus with an inflammatory substance. Their preclinical studies show that the presence of ADHD aggravates pain symptoms, while long-term pain does not affect ADHD-specific symptoms (attention symptoms, memory, and learning ability).

Our Spanish preclinical partner in the PiPCo study investigates inflammatory responses and changes in the intracellular signaling pathways in ADHD mice. They also tested a natural anti-inflammatory drug, “abscisic acid” (ABA), found in high concentrations in avocado and citrus fruits, among others. In previous animal studies, they showed that ABA administration prevented the pathological increase of inflammatory markers in the brain and that its effect resulted in improved cognitive capacity in different neurological animal models. This suggests that ABA is a potential candidate for treating inflammatory conditions of various etiologies to prevent declines in cognitive function.

## Discussion

The study has pragmatic and clear needs-oriented significance regarding the care and treatment of children and adolescents with ADHD and pain. The care of this patient group is currently undeveloped, and the existing level of knowledge is low. There are great socioeconomic gains to be made by streamlining and improving the care of this patient group. Studies examining the economic impact of ADHD in children/adolescents found annual national ADHD costs ranging between 1.041 and 1.529 billion euros, with ADHD-related costs of 9,860–14,483 euros per patient [50]. Here, we see the importance of early appropriate efforts and treatment to avoid unnecessary disability and persistence of psychiatric symptoms and pain.

The study also has a heavier scientific ambition—to contribute to the development of knowledge regarding the brain’s biological processes and mechanisms that drive and generate disability and suffering in the form of psychiatric symptoms and pain. The connections between neuroinflammation and psychiatric disorders are currently being studied intensively. Several recent studies imply a link between activation of the immune system and psychiatric symptoms [16, 18, 21–24]. In our study, we propose a method of studying a clinical manifestation of neuroinflammation in the form of pain sensitivity (i.e., central sensitization). We have a unique collaboration with physiotherapists who are experienced in the measurement of pain sensitivity in children. Together with our psychiatric reference framework and robust knowledge base, their knowledge creates a platform on which new knowledge can take shape.

We wish to present- based in reviewer criticism—a culture specific explanation, why we use the expression in this study “individuals with ADHD” and not “ADHD individuals”. In Sweden within clinics and ethical applications it is encouraged to use the expression “individuals with a …diagnose”, arguing that the disorder/diagnose does not identify/define a person. In our study population, only part of childhood ADHD diagnosis will persist into adulthood, and if it is correctly treated it should not leave any psychiatric or somatic/pain residues to adult hood.

We will likely not solve the whole puzzle of how inflammation and pain perception can affect mood and behavior, but we can identify pieces that, until today, have been undefined. By focusing on children and young individuals with ADHD and their special needs in connection with pain and healthcare encounters, we can assist this group in receiving the necessary attention. Perhaps, to some extent, we can also improve healthcare’s ability to manage and treat this population.

## Conclusions/clinical significance

The results will enable an improved understanding of the pathophysiology behind both ADHD and pain/central sensitization and may contribute to the development of new treatment strategies for pathological pain conditions in children and adolescents with ADHD. This project represents an important step in increasing awareness and providing opportunities for the treatment of attention and impulse control problems in children and adolescents with pain syndromes.

Because of the serious societal impact of this condition, it is crucial to raise awareness about the associations that exist between ADHD and other health conditions in order to enhance recognition and improve treatment.

The associations between pathological pain and neuropsychiatric diagnoses have rarely been studied in children and adolescents. We aim to contribute to an increased understanding of and attentiveness toward the specific needs of this patient group in healthcare encounters. Pain is common in healthcare settings but assessing and treating pain based on individual adaptations has not been commonly practiced. If our study confirms that children and adolescents with ADHD experience pain differently than those without, this knowledge is to be implemented in healthcare, both regarding the specialized care of chronic/pathological pain and ADHD and in everyday healthcare of children and young people with ADHD (e.g., in connection with more straightforward procedures that cause pain). Furthermore, if our study can point toward certain inflammatory processes that might underlie pain perception and ADHD, new anti-inflammatory or biological treatment opportunities may be possible.
